# Morphotypes and pigment profiles of halophilic bacteria: Practical data useful for novelty, taxonomic categorization and for describing novel species or new taxa

**DOI:** 10.1016/j.dib.2017.06.039

**Published:** 2017-06-30

**Authors:** Bhagwan N. Rekadwad, Chandrahasya N. Khobragade

**Affiliations:** a National Centre for Microbial Resource (NCMR), National Centre for Cell Science (NCCS), Pune, India; b School of Life Sciences, Swami Ramanand Teerth Marathwada University, Nanded, India

**Keywords:** Bergey׳s manual, Coastal region, Hydrocarbon degrader, Oil spill, Pigment producer, Taxonomic classification of bacteria

## Abstract

Halophilic bacteria were isolated from oil spill samples collected from West-coast of Goa. Bacteria were isolated from oil studded soil, salt marsh and offshore samples (A, A7, CSM, CB and CM) collected along the West coastline in Goa (India) i.e. Arambol beach, Calanguate beach, Candolim beach and Colva beach on Zobell Marine agar, R2A agar, Mannitol salt agar and Blood agar at temperature 22 to 24 °C. Isolates showed growth in the presence of hydrocarbons (1% phenanthrene and 2% bitumen). Diverse profiles of pigments were observed on different nutrient medium. Color of pigments produced on agar media recorded as per standard color chart. All isolates showed different growth pattern. Isolate no 11 (GOACSMMS-11) showed three different morphological features/growth patterns on Zobell Marine Agar and R2A medium in the presence of hydrocarbons. Results obtained yield new information which gives a clear idea about morphological features and pigmented profiles of hydrocarbon resistant morphotypes in the presence different media compositions. The presented datasets will be useful for studies on bacterial species showing high sequence similarity. Hence, generated data serves as a benchmark for to distinguish between genetically similar bacteria and for further research in phenotype based microbial diversity, microbial ecology of microorganisms and microbial systematics and taxonomy in addition to genotype data.

## Specifications Table

**Table:** 

Subject area	*Biology*
More specific subject area	*Microbiology*
Type of data	*Table, figure*
How data was acquired	*Visual, Microscope, Laboratory tests*
Data format	*Raw, analyzed*
Experimental factors	*Isolation and pure culture of microorganisms*
Experimental features	*Hydrocarbon (phenanthrine and bitumen) were used for the studies on morphological features of bacteria*
Data source location	*NCMR, NCCS, Pune (India)*
Data accessibility	*Data incorporated within this article*

## **Value of the data**

• Data is given in the paper help to describe the morphological features and diversity of bacteria.
• Data presented in this paper acts as key features for determining novelty of species if microorganism showing more genomic similarity i.e. for taxonomic categorization and classification of bacteria.
• Data generated serves as the benchmark for further research in microbial diversity, microbial ecology of microorganisms and microbial systematic and taxonomy.

## 1 Data

The data described in this paper highlights morphological features of halophilic bacteria (morphotypes). Bacterial species and their pigmented morphotypes were isolated from oil studded soil, salt marsh and offshore samples (A, A7, CSM, CB and CM) collected along the West coastline in Goa (India) i.e. Arambol beach, Calanguate beach, Candolim beach and Colva beach on Zobell Marine agar, R2A agar, Mannitol salt agar and Blood agar. Isolated bacterial colonies showing diverse morphological features were chosen for further study. Selected bacteria were sub-cultured and pure cultures are stored in refrigerator at 4 °C on continuous cycle. Isolated halophiles have optimum temperature 22 ± 2 °C. All isolates luxuriant growth in the presence of 20% salt concentration. Morphological features were recorded as per Bergey׳s Manual of Systematic Bacteriology and the International Code of Nomenclature of Bacteria (ICNB).

## 2 Experimental design, materials and methods

### 2.1 Isolation cultivation of microorganism

**Fig. 1:**
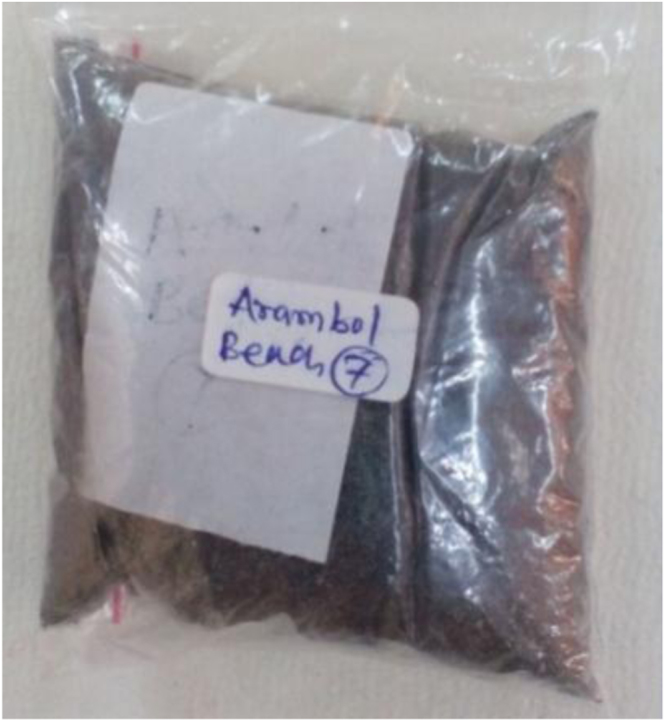
Soil sample collected from Arambol beach, North Goa.

Isolation of halophilic bacteria was carried out from samples- A, A7, CSM, CB and CM (approximately 100 g each)- collected from oil studded soil, salt marsh and offshore along West coastline in Goa (India) i.e. Arambol beach, Calanguate beach, Candolim beach and Colva beach (Fig. 1). Nineteen bacterial species were isolated on separately spread Zobell Marine agar, R2A agar, Mannitol salt agar and Blood agar with and without 1% phenanthrene in triplicates at temperature 22 °C [1], [2], [3], [4]. Selected bacterium was streaked on same media used in former step for obtaining bacterial cultures in pure form. Phenanthrene (1%) was dissolved in acetone (HiMedia, AR grade) while bitumen (2%) was dissolved in chloroform (HiMedia, AR grade). Phenanthrene and bitumen solutions were spread separately on plates in triplicates. Dissolving solvents were allowed evaporate at 40 °C aseptically. All halophilic bacteria were allowed to grow on Zobell Marine agar for confirmation of growth in the presence hydrocarbons- 1% phenanthrene and 2% bitumen- in the separate experiments. Zobell Marine Agar 2216 (M384) and R2A agar (M1743) were used as encrypt medium for cultivation and preservation of microorganisms in later experiments [5], [6], [7]. Medium M384 and M1743 were slightly modified and glycerol (4%) for preservation of bacteria at 4 °C. M384 medium was used for studies on morphological features and pigment production ability/tests. These hydrocarbon containing plates were used for isolation and cultivation of microorganisms in later experiments. Selected species were also checked for the production of pigments in Zobell Marine broth. All isolates incubated at 22 °C for 24–96 h. Results were recorded in lab notebook for the experiments (morphological features, biochemical tests, hydrocarbon tolerance/resistance and pigment production ability of isolated bacteria.

### 2.2 Interpretation of results as per obtained results shown table and figures

**Fig. 2:**
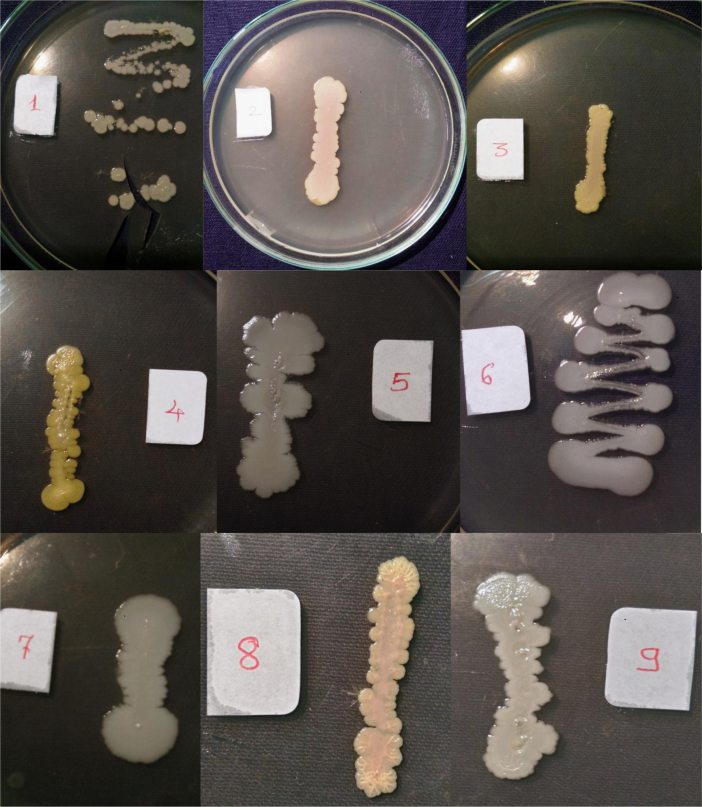

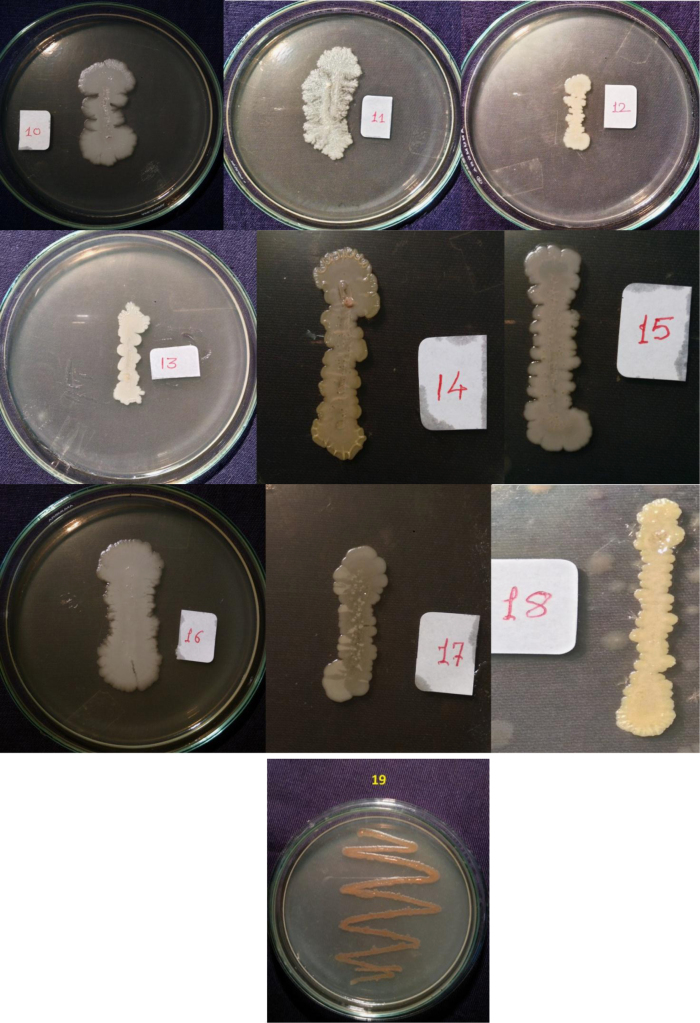
Phenotype of halophiles on medium M384 in the presence of 1% phenanthrene..

**Fig. 3:**
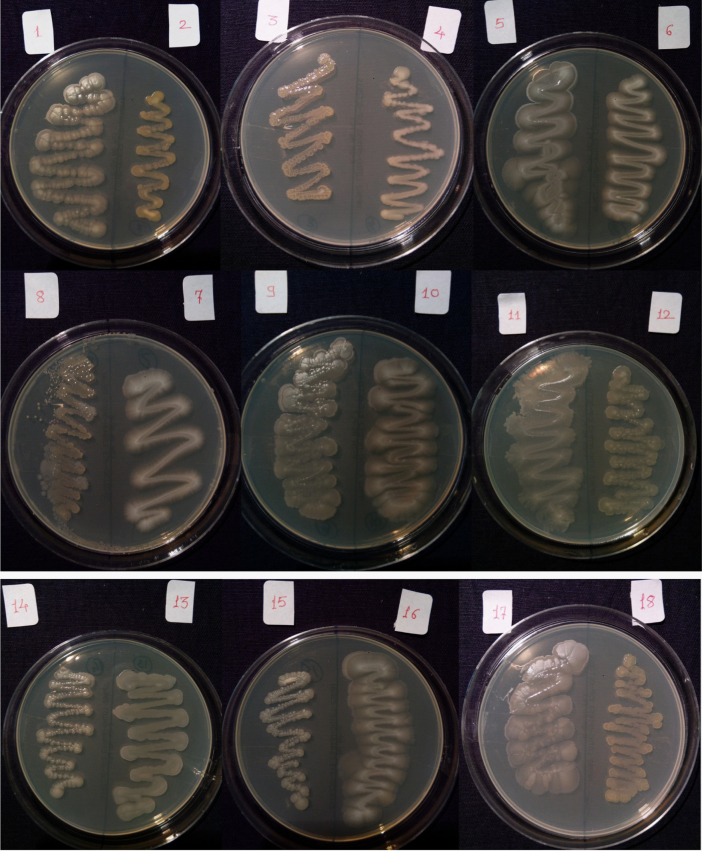
Phenotype of halophiles on medium M1743in the presence of 1% phenanthrene.

**Fig. 4:**
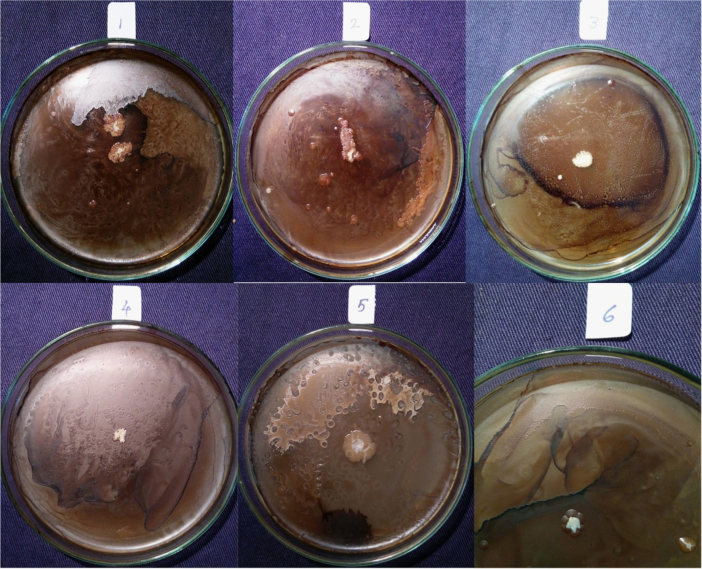

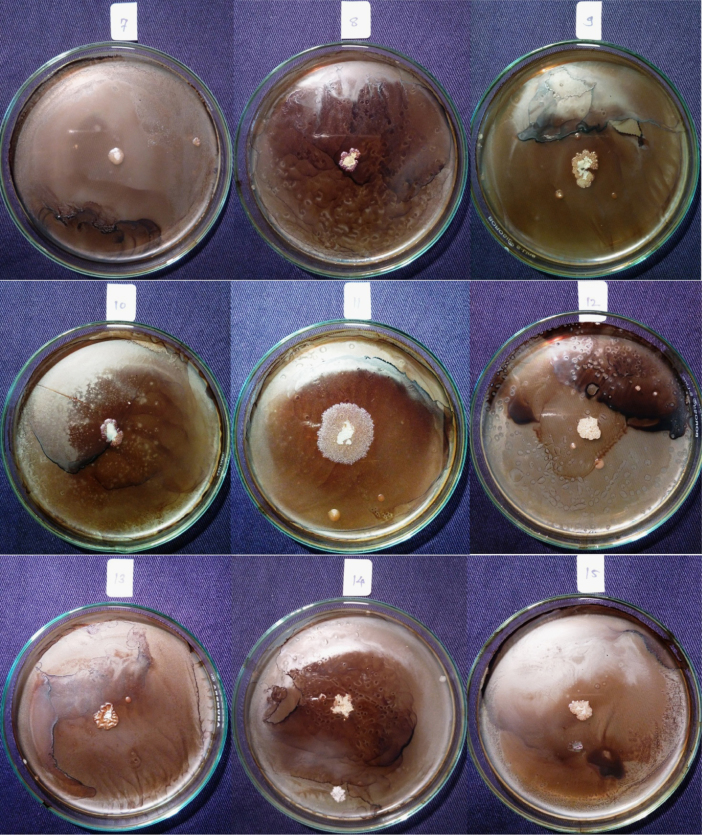

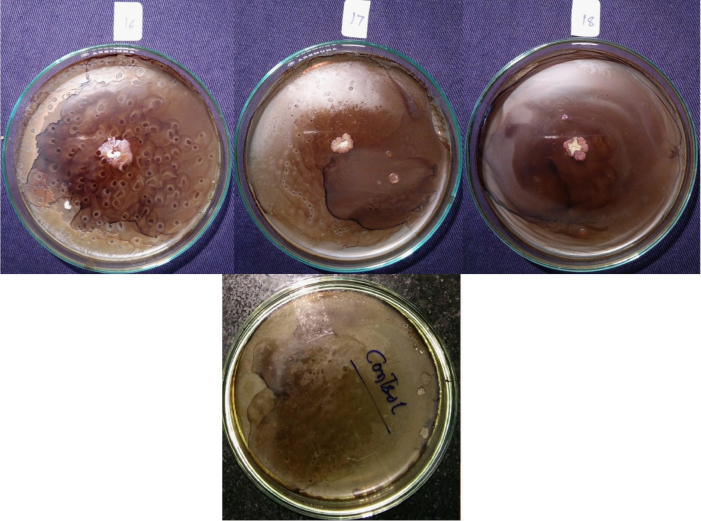
Phenotype of halophiles on medium M384 in presence of 2% Bitumen (1 mL).

**Fig. 5:**
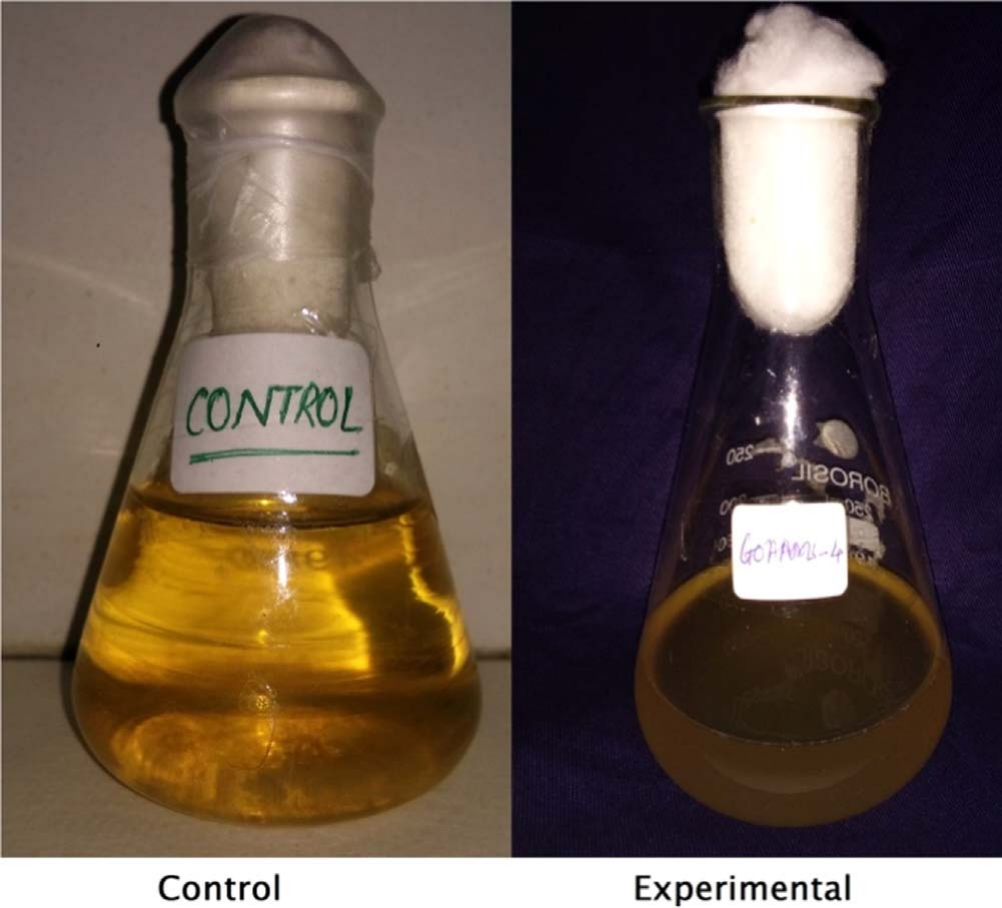
Yellow pigment produced by bacteria in Zobell Marine broth (M384).

Isolates showed growth in the presence of hydrocarbons (1% phenanthrene and 2% bitumen). Diverse profiles of pigments were observed on different medium. Color of pigments produced on agar media recorded as per standard color chart were Antique white, Misty rose, Papaya white, Ghost white, Gainsboro, Light Golden Rod, Moccasin, Lemon Chiffon, Ivory, Mint cream, White smoke, Light orange, Wheat, Floral white, Old lace, Pink etc. All isolates showed different growth pattern. Isolate no 11 (GOACSMMS-11) showed three different morphological growth patterns on Zobell Marine Agar and R2A medium in the absence and presence of hydrocarbons. Isolate no. 19 showed Pink color on Medium M384 (Table 1; Fig. 2, Fig. 3, Fig. 4, Fig. 5).

**Table 1:** Phenotypes of halophiles isolated from samples collected at Arambol beach, Calanguate beach, Candolim beach and Colva beach in Goa (India).

**Isolate code on plate**	**Strain designation for correct identity**	**Strain designation for convenience**	**Color of pigment produced on Zobell Marine Agar (Medium no. M384)**	**Color of pigment produced on R2A Agar (Medium no.)**	**Growth on 1% Phenanthrene containing agar plate**	**Growth on 2% Bitumen (1 mL) containing agar plate**
1	GOAAR2-1	BNR-1	Antique White 1	Gainsboro	Yes	Yes
2	GOAAR2A-2	BNR-2	Misty Rose 1	Light Golden Rod/Moccasin	Yes	Yes
3	GOAAR2A-3	BNR-3	Papayawhip/Wheat1	Light Orange	Yes	Yes
4	GOAAMS-4	BNR-4	Pale golden rod/Light golden rod1	Old Lace	Yes	Yes
5	GOAAMS-5	BNR-5	Ghost White	Ghost White	Yes	Yes
6	GOAA7MS-6	BNR-6	Ghost White	Ghost White	Yes	Yes
7	GOAARR2A-7	BNR-7	Floral White	Ghost White	Yes	Yes
8	GOAA7R2A-8	BNR-8	Light Golden Rod 1	Wheat	Yes	Yes
9	GOACSMR2A-9	BNR-9	Ghost White	White Smoke	Yes	Yes
10	COACSMMS-10	BNR-10	Ghost White	Ghost White/White Smoke	Yes	Yes
11	GOACSMMS-11	BNR-11	Mint Cream	Old Lace	Yes	Yes
12	GOACBR2A-12	BNR-12	Ivory 2	Old Lace	Yes	Yes
13	GOAAR2A-13	BNR-13	Ivory 2	Old Lace	Yes	Yes
14	GOAA7MS-14	BNR-14	Light Golden Rod 1	Old Lace	Yes	Yes
15	GOAA7R2A-15	BNR-15	Lemon Chiffon 1	White Smoke	Yes	Yes
16	GOACSMMS-16	BNR-16	Ghost White	Ghost White/White Smoke	Yes	Yes
17	GOAA7R2A-17	BNR-17	Floral white	White Smoke	Yes	Yes
18	GOAA7R2A-18	BNR-18	Wheat 1/Light orange	Light Orange	Yes	Yes
19	GOABTMNBNR-19	BNR-19	Pink	Pink	Yes	Yes

This phenotypic data will have use in future to distinguish genetically similar bacterial group such as *Bacillus* and *Bacillus-*like bacteria for describing novel species and new taxa as per **International Code of Nomenclature of Bacteria (ICNB) or Bacteriological Code (BC).**

## Appendix A Supplementary material

Video 1. Arambol Beach (North Goa): Oil stained sand indicating the severity of oil pollution at the West coast of, Goa.
